# MicroRNA-520f-3p inhibits proliferation of gastric cancer cells via targeting SOX9 and thereby inactivating Wnt signaling

**DOI:** 10.1038/s41598-020-63279-y

**Published:** 2020-04-10

**Authors:** Jian-qing Chen, Zhi-ping Huang, Hui-fen Li, Yang-liu Ou, Feng Huo, Liang-kai Hu

**Affiliations:** 1 Department of Gastroenterology, Shidong Hospital, Yangpu District, Shanghai, Anhui Medical University, 999 Shiguang Road, Shanghai, 200438 China; 2 Department of Hepatobiliary Surgery, General Hospital of Southern Theatre Command, 111 Liuhua Road, Guangzhou, 510010 China; 3 Department of Interventional, Eastern Hepatobiliary Surgery Hospital, Second Military Medical University, 225 Changhai Road, Shanghai, 200438 China; 4 Department of General Surgery, Changhai Hospital, Second Military Medical University, 168 Changhai Road, Shanghai, 200433 China

**Keywords:** Metastasis, Cell migration

## Abstract

MicroRNAs (miRNAs) are known to be important in a variety of cancer types. The specific expression and roles of miR-520f-3p in the context of gastric cancer (GC), however, remains unknown. Herein we determined miR-520f-3p expression to be significantly reduced in human GC cells compared to cells of the gastric epithelium, with comparable down-regulation also being evident in gastric cancer tissue samples and the low expression of this miRNA was positively correlated with features of more aggressive large tumor size (p = 0.019), depth of invasion (p = 0.008), and distant metastasis (p = 0.037). We further found that lower levels of miR-520f-3p corresponded with poorer GC patient overall (p = 0.003) and disease-free (p = 0.036) survival. When over-expressed in GC cells, miR-520f-3p was able to impair their growth, proliferation, and survival, instead leading to the induction of apoptosis. We further found that miR-520f-3p was able to bind the *SOX9* 3′-UTR, thereby negatively regulating its expression in GC cells. Consistent with this model, *SOX9* and miR-520f-3p expression were negatively correlated with one another in GC tissues. When SOX9 was upregulated, this was also able to abrogate miR-520f-3p-mediated inactivation of Wnt/β-catenin signaling. Together our findings thus suggest that miR-520f-3p can act to suppress GC progression, at least in part via suppressing *SOX9* expression and thus disrupting Wnt/β-catenin signaling. Our results thus highlight potential novel therapeutic targets in GC worthy of future investigation.

## Introduction

Gastric cancer (GC) is one of the deadliest forms of cancer in the world^1–3^, and it is highly prevalent in China where it is the second most common and third deadliest cancer subtype^4–6^. While there have been many complex and comprehensive analyses of GC conducted to date, the mechanisms governing the onset and progression of this disease remain incompletely understood, thus limiting patient access to efficacious treatment options. Thus, there is a clear need to explore the molecular mechanisms of GC development and progression so as to design better therapeutic regimens.

MicroRNAs (miRNAs), which are non-coding RNAs that are very short in length^7^, directly bind to target mRNA 3′-untranslated regions (3′UTRs), thereby suppressing translation and/or driving mRNA degradation^8,9^. A wide range of miRNAs are able to modulate tumor cell proliferation, migration, and invasion, as well as other biological processes^10–13^. Many miRNAs have also been shown to be expressed abnormally in GC in humans, wherein they can function in either an oncogenic or tumor suppressor role, controlling target gene expression in a tumor type-specific manner^14,15^. Wnt/β-catenin signaling serves as a key mediator of GC progression, with this signaling activity being closely linked to poorer patient prognosis and a higher grade of tumor malignancy^16^. Previous work suggests that SOX9 promotes epithelial-mesenchymal transition via the Hippo-YAP signaling pathway in gastric carcinoma cells^17^. Herein, we examined the potential role of miR-520f-3p in human GC progression by SOX9-Wnt/β-catenin axis.

## Materials and methods

### Tissue specimens

A total of 92 pairs of GC patient tumor and normal paracancerous tissue were obtained from patients that underwent surgery at the Changhai Hospital of the Second Military Medical University between January 2013 and October 2014. Patients enrolled in the present study had not undergone any preoperative systemic or local anti-tumor therapy. Following collection, pathological examination was used to confirm GC diagnosis, and samples were snap frozen prior to storage at −80 °C. All patients provided written informed consent to participate, and the Ethics Committee of Changhai Hospital, Second Military Medical University approved all human studies. Patient demographics and clinical findings are listed in Table S1.

### Cell culture

Both GC cell lines (HGC-27 and MKN-45) and the control GES-1 gastric epithelial line came from the Type Culture Collection of the Chinese Academy of Sciences. Cells were cultured at 37 °C with 5% CO2 in RPMI-1640 containing 20% FBS and antibiotics (Gibco, USA). For miRNA transfection, GC cells in the exponential phase were transfected with 100 nM of miR-520f-3p mimics or appropriate scrambled control RNA constructs (GeneCopoeia Ltd., iGeneBio) using Lipofectamine 2000 (Invitrogen). After 48 h cells were used for downstream studies.

We additionally established two sets of primary GC cells and primary gastric epithelium cells from two female stage III GC donors (aged 50 and 52) as previously described^18^. Briefly, the fresh GC tissues and surrounding normal epithelial tissues after surgery were dissected and placed in triple enzyme medium (collagenase, hyaluronidase, and DNase) for 1 h. A 70-mm nylon cell strainer (Becton Dickinson, Beijing, China) wasperformed to filter the cell suspensions, thereafter cultured in complete RPMI medium abandoned of Fibroblasts, immune cells and vascular endothelial cells.

### Cell transfection

miR-520f-3p mimics and controls (miR-NC) were produced by Gene Pharma (Shanghai, China) and transfected into cells using Lipofectamine 2000 (Invitrogen) for transfection based on provided protocols. Furthermore, the Sox9 stable overexpression vector pCMV6-AC-Sox9 (termed Sox9; 0.8 µg; Myhalic Biotechnology Co., Ltd., Wuhan, China,) and vector control (termed Vector; 0.8 µg; Myhalic Biotechnology Co., Ltd.) were transfected into MKN-45 and HGC-27 cells using Nucleofector® Program X-01 (Lonza Group, Ltd., Basel, Switzerland), according to the manufacturer’s protocol.

### qRT-PCR

Trizol (Invitrogen) was used for extracting total RNA, which was quantified using an ND-2000 spectrophotometer (Nano Drop Technologies, DE, USA). cDNA was generated using M-MLV (Promega, WI, USA), each RT reaction included 150 ng template RNA, 1 ul of cDNA and a pool of RT primers. A SYBR Premix Ex Taq™ Kits (TaKaRa, Tokyo, Japan) were used on an Applied Biosystems 7900HT platform (Thermo Fisher Scientific) for measuring relative gene expression. The reaction conditions of the qRT-PCR were as follows: 98 °C for 10 min, followed by 42 cycles of 3 sec at 98 °C and 30 sec at 55 °C. Normalization of miRNA and mRNA expression was conducted using U6 and GAPDH, respectively, and the 2^−∆∆Ct^ method was used to compare gene expression.

### Primers

miR-520f-3p F 5′-GTGCCTGTTGCGTCTC-3′,

R 5′-GAAAGCCTAGCCGTATTCG-3′;

SOX9 F 5′-AGGAAGTCGGTG AAGA ACGG-3′,

R 5′-CGCCTTGAAGATGGCGTTG-3′;

GAPDH F 5′-TGACTTCAA CAGCGACACCCA-3′,

R 5′-CACCCTGTTGCTGTAGCCAAA- 3′;

U6 F 5′-GACCGAGTGTAGCAAGG-3′,

R 5′-GTTCTTCCGAGAACATATAC-3′.

### Cell viability assay

The cell viability assay was performed as pervious before^18^. Briefly, Indicated 2 × 10^3^/per well GC cells were seeded in 96-well plates per well. According to the cell count kit (cck-8, Dojindo, Japan) manufacturer’s instructions, cell viability was detected. The CCK-8 optical density (OD) per well was defined at the wavelength of 550 nm.

### EdU assay of cell proliferation

The EdU assays were performed as pervious before^18^. Briefly, indicated 5 × 10^3^/per well GC cells were seeded in 24-well plates. The cells were stained with EdU. EdU ratio (edu/dapi _ 100%) was recorded under fluorescence microscope (Zeiss, 1:100 magnification). The 10 random views of each condition contain a total of 500 cells to calculate the EdU L ratio.

### Colony formation assay

For colony formation assay was performed as previous^18^, indicated 3×10^3^ GC cells were seeded in 10-cm cell culture dish cultured for 2 weeks. The colonies were then fixed, dyed, and counted.

### TUNEL staining assay

Indicated 5 × 10^3^/per well GC cells were seeded onto the 24-well plates at cells. The following assays were performed as previous^18^ and 500 cells from ten random views were included to count TUNEL ratio.

### Bioinformatic predictions and luciferase reporter assays

Target Scan^19^ was utilized as a means of predicting miR-520f-3p target mRNAs. The DNA regions coding wild-type (WT) Sox9 3′-untranslated region (targeted binding sites) and a mutant sequence (targeted binding site deletion) were inserted into a pmirGLO vector (Promega Corporation). HEK-293 cells with WT and mutant vectors using Lipofectamine^®^ 2000 (Invitrogen; Thermo Fisher Scientific, Inc.) were co-transfected with miR-520f-3p mimics and miR-NC. After 48 h transfection, was Dual-luciferase reporter assay system (Promega Corporation, Madison, WI, USA) were performed to define firefly luciferase activity normalized to *Renilla* luciferase activity, according to the manufacturer’s protocol.

### Western blotting

RIPA buffer (Beyotime Institute of Biotechnology) and BCA kit (Thermo Fisher Scientific) was used to lyse cells and quantify protein levels, respectively and 20 µg of each sample was separated through 10% SDS-PAGE prior to transfer to a PVDF membrane. These blots were then blocked with 5% non-fat milk for 2 h, after which they were probed overnight with antibodies specific forSox9(1:1000 dilution; cat. no. ab185230), β-catentin (1:5,000 dilution; cat. no. ab32572), cyclinD1 (1:5,00 dilution; cat. no. ab16663), c-Myc (1:1,000 dilution; cat. no. ab190026), Lambin B1 (1:1,000 dilution; cat. no. ab16048) and α-Tubulin (1:5,000 dilution; cat. no. ab210797) at 4 °C. Next, blots were incubated for 1 h with HRP-conjugated goat anti-mouse or anti-rabbit (1:5,000; Bioworld Technology, Inc.) secondary antibodies, and were then washed prior to development via enhanced chemiluminescence (Thermo Fisher Scientific) together with a ChemiDoc Imaging Platform (Bio-Rad). The isolation of nuclei was performed using a Nuclear Extraction Kit (KeyGEN Biotech, Jiangsu, China) according to the manufacturer’s manual. Lambin B1 was used as a loading control of nuclear protein fraction.

### TOP/FOP flash reporter assay

Plasmids encoding TOP or FOP flash along with appropriate TCF/LEF DNA binding sites came from Upstate Biotechnology (NY, USA). Cells were first plated into 24-well plates followed by Lipofectamine 2000-mediated transfection with these TOP Flash or FOP Flash constructs and 10 ng of the control pRL-TK Renilla luciferase vector (Promega). After 24 h, luciferase activity was measured as above and normalized to Renilla luciferase activity.

### Statistical analysis

All values are presented as the mean ± standard deviation. Significant differences were determined using GraphPad 5.0 (GraphPad Software, Inc., La Jolla, CA, USA). Student’s t-test was used to determine significant differences between two groups. One-way analysis of variance (ANOVA) was used to determine significant differences between multiple testing. Student-Newman-Keuls test was used as a post hoc test following ANOVA. The association between miR-520f-3p expression and Sox9 MRNA level in tumor tissues was by Linear Regression test. P < 0.05 was considered statistically significant difference. All experiments were repeated three times.

### Ethical approval and informed consent

The experiment is not involved in the animal experiments, so not applicable. All patients provided written informed consent to participate, and the Ethics Committee of Changhai Hospital, Second Military Medical University approved all human studies. The methods were carried out in accordance with the relevant guidelines and regulations. The experiments using human samples were approved by the Ethics Committee of Changhai Hospital of the Second Military Medical University and Declaration of Helsinki.

## Results

### Human GC samples exhibit decreased miR-520f-3p expression

**Figure 1:**
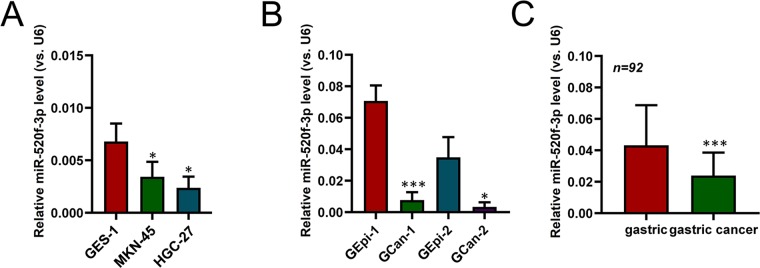
GC samples exhibit decreased miR-520f-3p expression. Human GC (A,**B**) or normal epithelium (**C**), miR-520f-3p expression was assessed via qRT-PCR, with U6 used for normalization (**A**–**C**). *P < 0.05; **P < 0.01; ***P < 0.001.

We first assessed the levels of miR-520f-3p present within human GC cells via qRT-PCR, revealing decreased levels of this miRNA in the GC cell lines (HGC-27 and MKN-45) relative to control GES-1 gastric epithelial line (Fig. 1A). We further found lower miR-520f-3p expression in patient-derived primary GC cells as compared to primary gastric epithelial cells (Fig. 1B). Consistent with this, miR-520f-3p expression was significantly reduced in gastric cancer tissues relative to surrounding normal gastric epithelial tissue (Fig. 1C). Thus, our findings indicate that human gastric cancer cells exhibit decreased miR-520f-3p expression.

### miR-520f-3p is associated with GC progression

We next extended our analyses to examine the relationship between miR-520f-3p expression and GC progression via dividing patients into miR-520f-3p-high and -low expressing groups (n = 73 and 72, respectively) based upon median expression levels of this miRNA. We then compared clinical findings in these patients, thus revealing a significant correlation between decreased expression of miR-520f-3p and large tumor size (p = 0.019), depth of invasion (p = 0.008), and distant metastasis (p = 0.037) (Table 1).

**Table 1:** Association between miR-520f-3p expression and clinicopathological features of human GC.

Clinical features	Total	miR-520f-3p		*p*-value
		High	Low	
		(N = 46)	(N = 46)	
Age (years)				0.650
<60	28	13	15	
≥ 60	64	33	31	
Gender				0.400
Male	52	28	24	
Female	40	18	22	
Tumor size (cm)				0.019
<5	37	24	13	
≥ 5	55	22	33	
Differentiation grade				0.825
Well	31	16	15	
Moderate + Poor	61	30	31	
TNM stage				0.388
I + II	58	27	31	
III	34	19	15	
Depth of invasion				0.008
T1 + T2	44	27	17	
T3 + T4	48	19	29	
Lymph node metastasis				0.804
No	58	25	33	
Yes	34	21	13	
Distant metastasis				0.037
No	72	41	31	
Yes	20	5	15	
CEA, µg/ml				0.676
<4.5	44	21	23	
≥ 4.5	48	25	23	
CA19-9, kU/L				0.143
<40	70	38	32	
≥ 40	22	8	14	

CA19-9 carbohydrate antigen 19-9; CEA, carcinoembryonic antigen; Pearson chi-square test was used for comparison between subgroups.

### Decreased expression of miR-520f-3p corresponds to poorer patient outcomes

**Figure 2:**
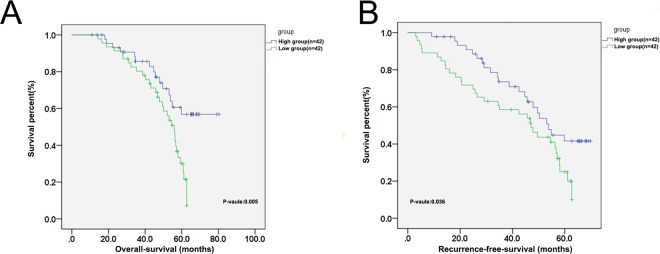
Decreased expression of miR-520f-3p corresponds to poorer patient outcomes. (**A**) OS was decreased in GC patients expressing lower levels of miR-520f-3p relative to those expressing higher levels of this miRNA. (BF) DFS was decreased in GC patients expressing lower levels of miR-520f-3p relative to those expressing higher levels of this miRNA.

We next analyzed the prognostic relevance of miR-520f-3p levels in this GC patient cohort. We found that miR-520f-3p-low patients exhibited a significantly shorter median overall survival (OS) compared to miR-520f-3p group (P = 0.003, Fig. 2A). Similar findings were also observed with respect to patient disease-free survival (DFS), in the -low and -high expressor groups, respectively (P = 0.036, Fig. 2B). This thus suggested that low expression of miR-520f-3p is negatively correlated with patient prognosis such that decreased expression of this miRNA is linked with poorer patient outcomes. In an additional multivariate analysis (Table 2), we confirmed a significant relationship between the expression of miR-520f-3p and GC patient OS (HR = 1.946, 95% CI: 1.256–2.678, p = 0.031) and DFS (HR = 1.632, 95% CI: 1.292–2.867, p = 0.038).

**Table 2:** Univariate and multivariate Cox regression analyses of overall survival and disease-free survival in GC patients.

Variables	Univariate analyses			Multivariate analyses		
	Hazard ratio	95% CI	P	Hazard ratio	95% CI	P
**Overall survival**						
Age (years) ≥ 60/<60	0.805	0.638–1.169	0.881	—	—	—
Gender Male/female	0.942	0.596–1.283	0.755	—	—	—
Tumor size (cm) ≥ 5/<5	1.753	1.279–2.861	0.018	2.097	1.192–2.252	0.036
Differentiation grade Poor+ moderate/well	3.134	1.369–3.693	0.007	2.013	1.145–2.598	0.016
TNM stage III/I + II	1.547	0.546–2.286	0.398	—	—	—
Depth of invasion pT3-4/pT1-2	1.783	0.693–3.810	0.332	—	—	—
Lymph node metastasis Yes/no	2.842	1.361–2.578	0.033	2.333	1.567–2.679	0.045
Distant metastasis Yes/no	3.161	1.527–3.967	0.013	1.783	0.574–2.788	0.357
CEA, µg/ml ≥ 4.5/<4.5	1.807	0.837–3.835	0.273	—	—	—
CA19-9, kU/L ≥ 40/<40	1.655	0.973–3.691	0.803	—	—	—
MiR-520f-3p expression low/high	2.31	1.524–3.429	0.025	1.946	1.256–2.678	0.031
**Disease-free survival**						
Age (years) ≥ 60/<60	0.854	0.525–1.809	0.624	—	—	—
Gender Male/female	0.742	0.739–1.507	0.203	—	—	—
Tumor size (cm) ≥ 5/<5	1.265	0.562-2.952	0.815	—	—	—
Differentiation grade Poor+ moderate/well	3.798	1.337–3.531	0.034	1.525	1.545–2.852	0.041
TNM stage III/I + II	1.891	0.818–2.437	0.825	—	—	—
Depth of invasion pT3-4/pT1-2	1.827	1.236–2.099	0.017	1.901	1.311–2.633	0.027
Lymph node metastasis Yes/no	3.272	1.433–4.629	0.018	1.587	1.211–2.094	0.021
Distant metastasis Yes/no	2.357	1.307–3.752	0.015	2.464	1.238–2.359	0.047
CEA, µg/ml ≥ 4.5/<4.5	1.676	0.804–1.122	0.107	—	—	—
CA19-9, kU/L ≥ 40/<40	1.423	0.457–1.669	0.438	—	—	—
MiR-520f-3p expression low/high	2.225	1.896–3.833	0.013	1.876	1.292–2.867	0.039

CA19-9 carbohydrate antigen 19-9; HR, hazard ratio; 95% CI, 95% confidence interval.

### Overexpression of miR-520f-3p impairs the growth of gastric cancer cells

**Figure 3:**
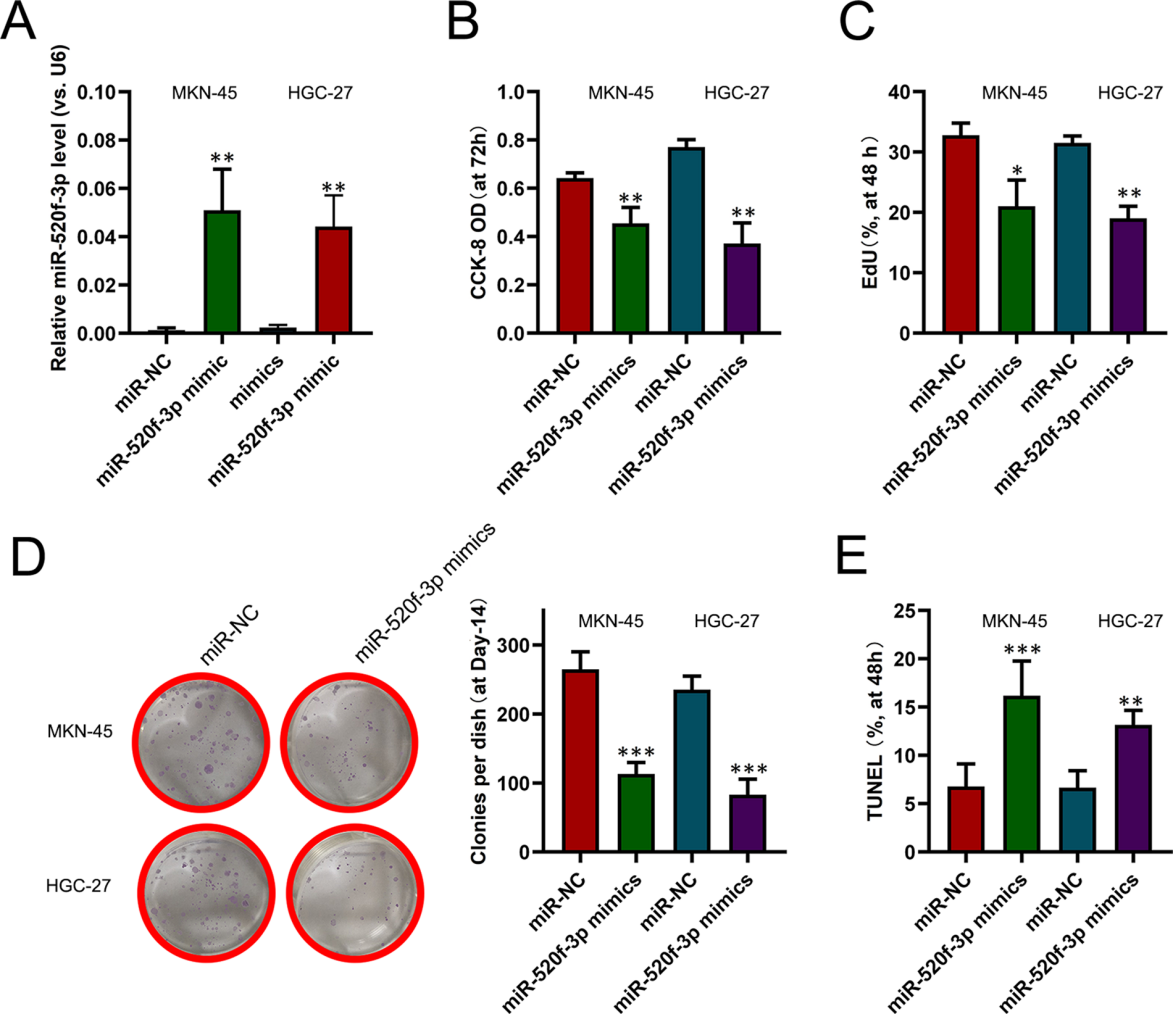
miR-520f-3p inhibits GC cell growth. HGC-27 and MKN-45 cells underwent transfection using a miR-520f-3p mimic or negative control (NC) construct, and then after 48 hours miR-520f-3p expression was assessed via qRT-PCR (**A**); Cell proliferation (**B**–**D**) and apoptosis (**E**) were measured, using the same number of cells for initial seeding conditions in all assay. *P < 0.05; **P < 0.01; ***P < 0.001.

To functionally characterize miR-520f-3p in GC cells, HGC-27 and MKN-45 cells were transfected with miR-520f-3p mimics or control constructs (Fig. 3A). CCK8 and EdU incorporation assay revealed miR-520f-3p overexpression to be associated with a significantly suppression of HGC-27 and MKN-45 cell proliferation (Fig. 3B,C), as was colony formation (Fig. 3D). TUNEL staining revealed a significant increase in the rates of apoptosis in HGC-27 and MKN-45 cells with elevated miR-520f-3p expression (Fig. 3E).

### miR-520f-3p targets the *SOX9* mRNA

**Figure 4:**
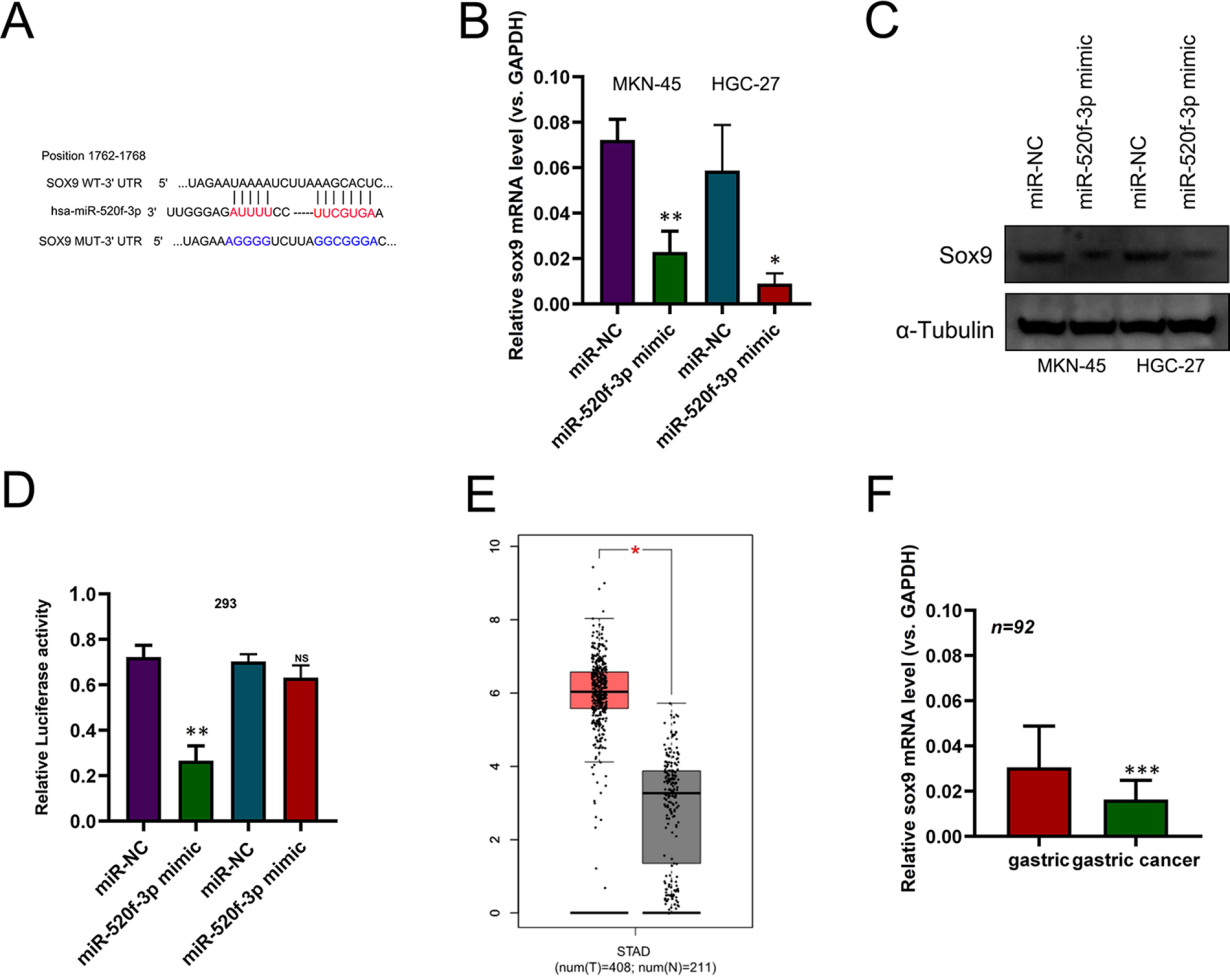
miR-520f-3p directly targets SOX9 in GC. The alignment of the miR-520f-3p sequence with that of predicted SOX9 binding sites. (**B**) SOX9 expression was assessed via qRT-PCR in HGC-27 and MKN-45 cells following miR-520f-3p or miR-NC transfection. (**C**) SOX9 levels were assessed via Western blotting in HGC-27 and MKN-45 cells following miR-520f-3p or miR-NC transfection (**D**) Dual-luciferase reporter assays were used to reveal a reduction in luminescence upon miR-520f-3p mimic co-transfection with the SOX9-Wt vector, without a comparable effect for the SOX9-mut vector in 293 cells. (**E**) GEPIA data indicated that SOX9 was increased in GC. (**F**) Measurement of SOX9 expression via qRT-PCR in 92 paired GC and normal gastric epithelial tissue samples. *P < 0.05; **P < 0.01; ***P < 0.001.

We next assessed the molecular mechanisms whereby miR-520f-3p influences GC, using bioinformatics tools in order to predict target genes corresponding to this miRNA. Through this analysis we were able to identify *SOX9* as a putative miR-520f-3p target (Fig. 4A). The mRNA and protein expression levels of SOX9 were markedly reduced in HGC-27 and MKN-45 cells transfected with miR-520f-3p mimics (Fig. 4B,C). We then used the dual-luciferase reporter assay to confirm whether miR-520f-3p was able to directly bind the SOX9 3′-UTR. Our results showed that when compared to the miR-NC group, miR-520f-3p mimics could significantly attenuate the luciferase activity of 293 cells driven by SOX9-WT, but not by SOX9-MUT (Fig. 4D). From GEPIA (Gene Expression Profiling Interactive Analysis) data indicated that SOX9 was increased in GC compared to normal tissues (Fold-change >2, P < 0.001, Fig. 4E). We further used qRT-PCR to assess *SOX9* expression in 92 paired GC and normal gastric epithelial tissue samples, revealing a significant increase in *SOX9* levels in the tumor samples relative to matched normal controls (Fig. 4F). In addition, *SOX9* and miR-520f-3p expression were significantly reversely correlated with one another in gastric cancer tissues (Spearman’s r = −0.5626, P < 0.05). Thus, these findings therefore indicated that *SOX9* is a direct miR-520f-3p target.

### miR-520f-3p targeting of SOX9 inhibits GC cell Wnt/β-catenin signaling

**Figure 5:**
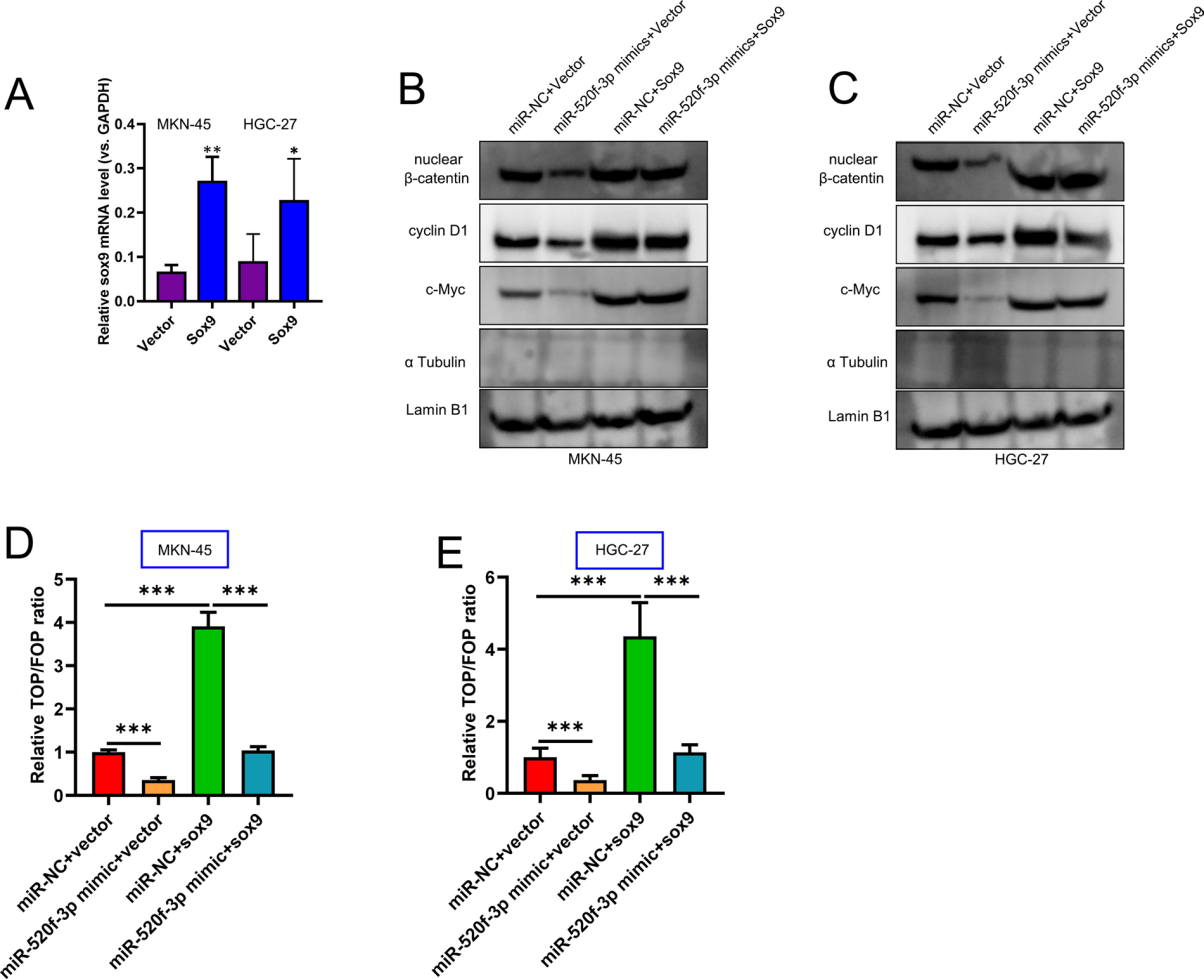
SOX9 upregulation overcomes miR-520f-3p-mediated disruptions to Wnt/β-catenin signaling. (**A**) qRT-PCR analysis of SOX9 mRNA expression after transfection in HGC-27 and MKN-45 cells. (**B**,**C**) How miR-520f-3p or SOX9 influenced nuclear β-catenin, c-myc, and cyclin D1 levels were assessed via Western blotting.Alpha tubulin as a negative control. (**D**,**E**) The TOP/FOP luciferase assay was utilized as a means of assessing how miR-520f-3p and SOX9 influenced Wnt/β-catenin signaling. *P < 0.05; **P < 0.01; ***P < 0.001.

We next explored the potential ability of miR-520f-3p to modulate Wnt/β-catenin signaling through its influence on the expression of SOX9. Firstly, over-expression of SOX9 was performed to conduct functional studies in HGC-27 and MKN-45 cells (Fig. 5A). We found that when overexpressed, miR-520f-3p was able to markedly suppress β-catenin nuclear localization, and this coincided with decreased cyclin D1 and c-myc levels, whereas when SOX9 expression was increased these effects were reversed (Fig. 5B,C). In contrast, when SOX9 expression was up-regulated, nuclear β-catenin, c-myc, and cyclin D1 levels rose, and miR-520f-3p co-transfection reversed this upregulation to a significant degree (Fig. 5D,E). Using a TOP/FOP luciferase activity assay, we were further able to provide clear evidence that miR-520f-3p overexpression suppressed Wnt/β-catenin signaling, and elevated SOX9 expression reversed this, with the converse also being true with miR-520f-3p suppressing to observed enhancement of Wnt/β-catenin signaling in cells upregulating SOX9 (Fig. 5C,D). Thus, this demonstrated that miR-520f-3p is able to suppress Wnt/β-catenin signaling via inhibiting SOX9.

## Discussion

GC was one of deadliest malignant tumors in the world^1–3^. Amounts of studies have highlighted a key role for abnormal miRNA expression in GC development^19^. Many researchers have recently made progress in the use of miRNA-based cancer therapeutic strategies in order to specifically target genes known to drive tumor proliferation, survival, or metastasis, thereby allowing for the control of tumor effector functionality and growth^14,15^. In our study we found that this miRNA was expressed at lower levels in GC patient tumors tissues and cell lines. In addition, we found miR-520f-3p down-regulation to correlate with features of more advanced GC including lymph node metastasis (p = 0.016), depth of invasion (p = 0.007), and distant metastasis (p = 0.033), suggesting that this miRNA is associated with poorer patient outcomes, as was confirmed by an analysis revealing that GC patients expressing low levels of miR-520f-3p have shorter OS and DFS than did patients expressing low levels of this miRNA. A multivariate analysis additionally confirmed that miR-520f-3p expression was an independent predictor of GC patient OS and DFS. Through *in vitro* analyses we further found that miR-520f-3p was able to inhibit GC cell proliferation, indicating it is important for regulating cancer cell growth.

miRNAs are known to control gene expression via target mRNA 3′-UTR binding. To assess how miR-520f-3p affects GC, we therefore sought to identify its target genes in GC cell lines. Through a bioinformatics prediction, we identified SOX9 as a target of this miRNA, and we chose to validate this finding based on previous reports indicating that SOX9 is known to be a key regulator of GC development and progression^20^. Multiple miRNAs have been shown to target SOX9, such as miR-124^21^, miR-206^22^, and miR-32^23^. We were able to use qRT-PCR, western blotting, and luciferase reporter assays to confirm that miR-520f-3p directly bound to and regulated SOX9 mRNA expression. We further determined that SOX9 and miR-520f-3p expression were negatively correlated with one another in GC tissues. Together, our results thus provide clear evidence that miR-520f-3p may influence GC progression at least in part via suppressing SOX9 expression. Previous studies indicated that SOX9 played key role in GC progression^17,24–26^. As such, this suggests the possibility that targeted therapies modulating the miR-520f-3p/SOX9 pathway could offer novel opportunities to improve gastric cancer patient survival.

Wnt/β-catenin signaling is important for regulating tumor invasion, proliferation, angiogenesis, and metastasis, thereby promoting disease progression^27^. In this context of cancer development, SOX9 expression is also known to be directly linked to Wnt/β-catenin signaling in GC^17^. Herein we found that overexpression of miR-520f-3p disrupted Wnt/β-catenin signaling, whereas the opposite was true when SOX9 was overexpressed. These results thus showed that miR-520f-3p can target SOX9, thereby inactivating Wnt/β-catenin signaling and disrupting GC cell growth. However, the effect of Sox9 knockdown in GC cells is lacked in the study. Further studies are needed to confirm that whether down-regulation of SOX9 produced a similar phenotype.

In summary, we found that miR-520f-3p expression is reduced in the context of GC in human tissue samples and cells. Overexpression of miR-520f-3p in GC cells markedly disrupted their ability to proliferate, likely at least in part via targeting SOX9. Our results thus highlight potential novel therapeutic strategies for treating GC. Our future research efforts will focus on further exploring their clinical and diagnostic significance to further understand the optimal clinical utility for these markers of GC.

## Supplementary information

Supplementary information

## Data Availability

All data generated or analyzed during this study are included in this published article.

## References

[1] Chen W (2015). Cancer statistics in China. Ca Cancer J. Clin..

[2] Siegel, R. L. *et al*. Cancer statistics for Hispanics/Latinos, 2015. *Ca Cancer J Clin* **65**, 457–480 (2015).

[3] Siegel RL, Miller KD, Jemal A (2017). Cancer statistics, 2017. Ca A Cancer J. Clinicians.

[4] Freddie B (2018). Global Cancer Statistics 2018: GLOBOCAN Estimates of Incidence and Mortality Worldwide for 36 Cancers in 185 Countries. Ca A Cancer J. Clinicians.

[5] Hartgrink HH, Jansen EPM, Grieken NCTV, Velde CJHVD (2009). Gastric cancer..

[6] Ferlay J (2019). Estimating the global cancer incidence and mortality in 2018: GLOBOCAN sources and methods. Int. J. Cancer.

[7] Kim VN (2005). MicroRNA biogenesis: coordinated cropping and dicing. Nat. reviews. Mol. Cell Biol..

[8] Bartel DP (2009). MicroRNAs: Target Recognition and Regulatory Functions. Cell.

[9] Calin GA, Croce CM (2006). MicroRNA signatures in human cancers. Nat. Rev. Cancer.

[10] Aigner, A. MicroRNAs (miRNAs) in cancer invasion and metastasis: therapeutic approaches based on metastasis-related miRNAs. **89**, 445–457 (2011). 10.1007/s00109-010-0716-0 21234533

[11] Cho WCS (2010). MicroRNAs: Potential biomarkers for cancer diagnosis, prognosis and targets for therapy. Int. J. Biochem. Cell Biol..

[12] Nikolouzakis T (2018). Improving diagnosis, prognosis and prediction by using biomarkers in CRC patients (Review). Oncol. Rep..

[13] Rottiers V, Nr AM (2012). MicroRNAs in metabolism and metabolic disorders. Nat. reviews. Mol. Cell Biol..

[14] Ke J (2019). MiR-1-3p suppresses cell proliferation and invasion and targets STC2 in gastric cancer. Eur. Rev. Med. Pharmacol. Sci..

[15] Liu, W. L., Wang, H. X., Shi, C. X., Shi, F. Y. & Zhao, L. Y. MicroRNA-1269 promotes cell proliferation via the AKT signaling pathway by targeting RASSF9 in human gastric cancer. **19**, 308, 10.1186/s12935-019-1026-4 (2019). 10.1186/s12935-019-1026-4 PMC6873743 31768130

[16] Liu G (2019). GJB4 promotes gastric cancer cell proliferation and migration via Wnt/CTNNB1 pathway. OncoTargets Ther..

[17] Zhou H, Li G, Huang S, Feng Y, Zhou A (2019). SOX9 promotes epithelial-mesenchymal transition via the Hippo-YAP signaling pathway in gastric carcinoma cells. Oncol. Lett..

[18] Bing, Z., Hui-Yu, L., Yun-Hong, X., Ai-Gui, J. & Yu-Xin, L. Long non-coding RNA EPIC1 promotes human lung cancer cell growth. *Biochemical and Biophysical Research Communications* (2018). 10.1016/j.bbrc.2018.07.046 30029875

[19] Shrestha S (2014). A systematic review of microRNA expression profiling studies in human gastric cancer. Cancer Med..

[20] Ren X (2015). PPARγ suppressed Wnt/β-catenin signaling pathway and its downstream effector SOX9 expression in gastric cancer cells. Med. Oncol..

[21] Wang X (2016). miR-124 inhibits cell proliferation, migration and invasion by directly targeting SOX9 in lung adenocarcinoma. Oncol. Rep..

[22] Zhang, Y. J. *et al*. miR-206 inhibits non small cell lung cancer cell proliferation and invasion by targeting SOX9. **8**, 9107–9113 (2015). PMC4538070 26309565

[23] Zhu D, Chen H, Yang X, Chen W, Yu L (2015). miR-32 functions as a tumor suppressor and directly targets SOX9 in human non-small cell lung cancer. Oncotargets Ther..

[24] Carrasco-Garcia E (2019). Therapeutic relevance of SOX9 stem cell factor in gastric cancer. Expert. Opin. Therapeutic Targets.

[25] Song, S. *et al*. PPARdelta Interacts with the Hippo Coactivator YAP1 to Promote SOX9 Expression and Gastric Cancer Progression, 10.1158/1541-7786.mcr-19-0895 (2019). 10.1158/1541-7786.MCR-19-0895 31796534

[26] Xue M (2019). MicroRNA-613 induces the sensitivity of gastric cancer cells to cisplatin through targeting SOX9 expression. Am. J. Transl. Res..

[27] He L (2019). Wnt/β-catenin signaling cascade: A promising target for glioma therapy. J. Cell. Physiol..

