# Supplementary material for: MicroRNA-520f-3p inhibits proliferation of gastric cancer cells via targeting SOX9 and thereby inactivating Wnt signaling
Source: Sci Rep. 2020 Apr 10;10:6197. doi: 10.1038/s41598-020-63279-y (PMC7148374; doi:10.1038/s41598-020-63279-y)

**MicroRNA-520f-3p inhibits proliferation of gastric cancer cells via targeting SOX9 and thereby inactivating Wnt signaling**

Jian-qing Chen1*, Zhi-ping Huang2,3*, Hui-fen Li3, Yang-liu Ou4, Feng Huo2#, Liang-kai Hu1#

1 Department of Digestive Internal, Shidong Hospital, Yangpu District, Shanghai, Anhui Medical University, 999 Shiguang Road, Shanghai, 200438, China.

2 Department of Hepatobiliary Surgery, General Hospital of Southern Theatre Command, 111 Liuhua Road, Guangzhou, 510010, China.

3 Department of Interventional, Eastern Hepatobiliary Surgery Hospital, Second Military Medical University, 225 Changhai Road, Shanghai, 200438, China.

4 Department of General Surgery,Changhai Hospital, Second Military Medical University, 168 Changhai Road, Shanghai, 200433, China.

*Contribution equality

#Correspondence to:

Feng Huo, email: gzhuof@163.com

Liang-kai Hu, email: huliangkai800116@163.com

**Table S1. The clinicopathological features of 92 human GC**

| **Clinical features** | **N** |
| --- | --- |
|  |  |
|  |  |
| **Age (years)** |  |
| < 60 | 28 |
| ≥ 60 | 64 |
| **Gender** |  |
| Male | 52 |
| Female | 40 |
| **Tumor size (cm)** |  |
| < 5 | 37 |
| ≥ 5 | 55 |
| **Differentiation grade** |  |
| Well | 31 |
| Moderate + Poor | 61 |
| **TNM stage** |  |
| I + II | 58 |
| III | 34 |
| **Depth of invasion** |  |
| T1 + T2 | 44 |
| T3 + T4 | 48 |
| **Lymph node metastasis** |  |
| No | 58 |
| Yes | 34 |
| **Distant metastasis** |  |
| No | 72 |
| Yes | 20 |
| **CEA,** **µg/ml** |  |
| < 4.5 | 44 |
| ≥ 4.5 | 48 |
| **CA19-9, kU/L** |  |
| < 40 | 70 |
| ≥ 40 | 22 |

CA19-9 carbohydrate antigen 19-9; CEA, carcinoembryonic antigen; Pearson chi-square test was used for comparison between subgroups.

**Supplement FigureS1**

**Uncropped images of blots and gels in the article.**


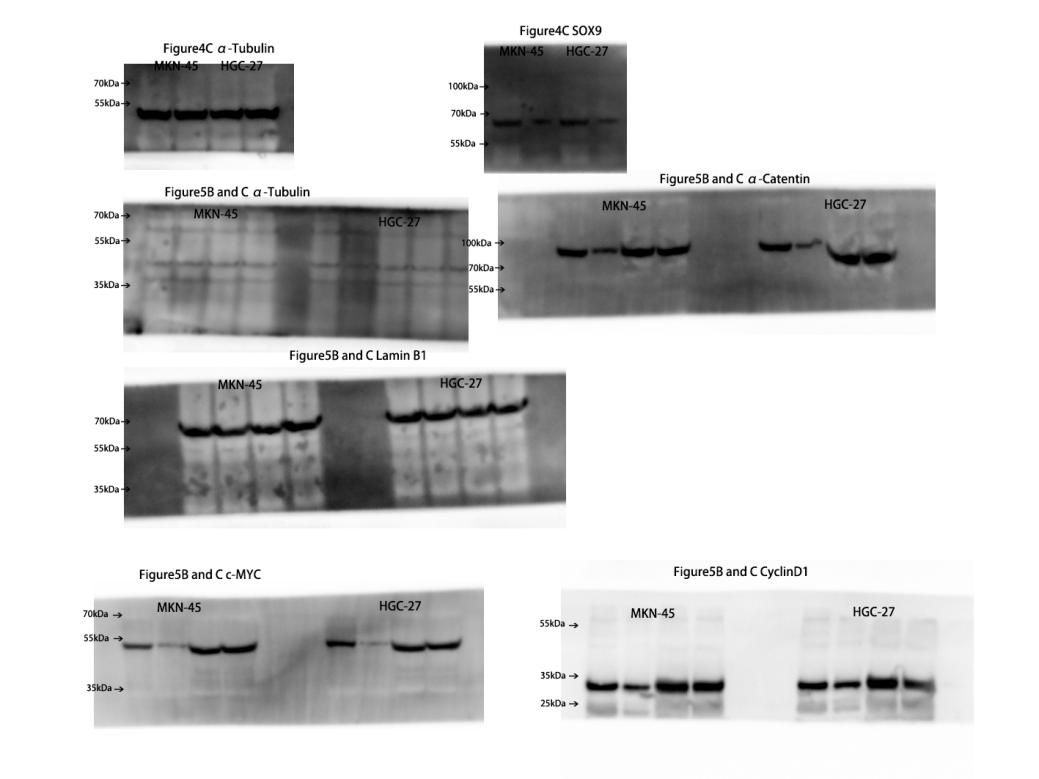

Supplement: Supplementary file 1 — Supplementary information [file 41598_2020_63279_MOESM1_ESM.docx]
